# Strain Monitoring and Crack Detection in Masonry Walls under In-Plane Shear Loading Using Smart Bricks: First Results from Experimental Tests and Numerical Simulations †

**DOI:** 10.3390/s23042211

**Published:** 2023-02-16

**Authors:** Andrea Meoni, Antonella D’Alessandro, Felice Saviano, Gian Piero Lignola, Fulvio Parisi, Filippo Ubertini

**Affiliations:** 1 Department of Civil and Environmental Engineering, University of Perugia, Via G. Duranti 93, 06125 Perugia, Italy; 2 Department of Structures for Engineering and Architecture, University of Naples “Federico II”, Via Claudio 21, 80125 Naples, Italy

**Keywords:** smart bricks, masonry structures, structural health monitoring, smart materials, self-sensing structural materials, diagonal compression tests, strain measurements, crack detection, shear-induced damage

## Abstract

A diffuse and continuous monitoring of the in-service structural response of buildings can allow for the early identification of the formation of cracks and collapse mechanisms before the occurrence of severe consequences. In the case of existing masonry constructions, the implementation of tailored Structural Health Monitoring (SHM) systems appears quite significant, given their well-known susceptibility to brittle failures. Recently, a new sensing technology based on smart bricks, i.e., piezoresistive brick-like sensors, was proposed in the literature for the SHM of masonry constructions. Smart bricks can be integrated within masonry to monitor strain and detect cracks. At present, the effectiveness of smart bricks has been proven in different structural settings. This paper contributes to the research by investigating the strain-sensitivity of smart bricks of standard dimensions when inserted in masonry walls subjected to in-plane shear loading. Real-scale masonry walls instrumented with smart bricks and displacement sensors were tested under diagonal compression, and numerical simulations were conducted to interpret the experimental results. At peak condition, numerical models provided comparable strain values to those of smart bricks, i.e., approximately equal to 10^−4^, with similar trends. Overall, the effectiveness of smart bricks in strain monitoring and crack detection is demonstrated.

## 1. Introduction

To enhance the safety of new and existing buildings, as well as that of their occupants, Structural Health Monitoring (SHM) systems can be used to assess the structural performance of constructions in real-time [1,2]. The diffuse and continuous monitoring of the in-service structural response of a building can allow for the identification of early modifications to the monitored parameters, such as, among others, strains, accelerations, and displacements, which may be indicative of the incipient formation of crack paths and collapse mechanisms before the occurrence of severe consequences [3,4]. Among the various structural typologies, existing masonry constructions are particularly susceptible to brittle damage mechanisms; this is mainly due to their structural design, which commonly accounts only for static loads, and the intrinsic heterogeneity of masonry, which often results in a complex mechanical behavior that is difficult to predict [5,6,7]. Furthermore, in the case of historic structures, material degradation due to aging can lead to a loss of strength with a consequent reduction in structural capacity to service loads and critical solicitations, such as those experienced during seismic events [8,9,10]. Considering this, it appears evident that the implementation of tailored SHM systems to masonry constructions can benefit the preservation of the built heritage and human lives. The monitoring approaches that are usually applied to masonry constructions are both vibration- and strain-based SHM methods; the monitoring of displacements is also widely adopted in common practice, especially to assess crack growth over time [11,12]. The potential applications of vibration-based SHM methods encompass the monitoring of slender structures, such as historic masonry towers [13,14,15] and buildings, such as palaces and churches [16,17,18], and bridges [19,20,21]. The practical applications of strain-based SHM methods to similar structural settings can be found in the literature [22,23,24,25,26]. Computer vision-based SHM approaches are also frequent in the literature, with interesting applications to masonry structures and more [27,28,29,30,31,32]. Although such techniques appear to be promising, the practical applications of these methods are still limited in number compared to traditional monitoring approaches. Despite their maturity, the sensing technologies available on the market, such as accelerometers, strain gauges, fiber optic sensors, and Linear Variable Differential Transformers (LVDTs), to cite a few of the most used, have shown some drawbacks in terms of SHM applications. High costs, low durability over time, and difficulties in diffuse deployment and external installation are among the most common issues experienced in practical applications, which often limit the extensive utilization of these sensing devices [33,34]. Furthermore, traditional sensing technologies possess different physical and mechanical properties to those of the monitored structures, a circumstance that may lead to an unrealistic assessment of the magnitude of the monitored parameters [35]. Recently, an innovative sensing technology tailored to monitoring strain in masonry constructions, named smart brick, was proposed in the literature. Smart bricks resemble conventional clay bricks; however, they are able to self-sense modifications in their strain state [36]. Accordingly, these innovative brick-like sensors can be embedded within masonry load-bearing structures, giving rise to durable and diffuse SHM systems [37]. At present, the SHM applications of smart bricks encompass (i) the monitoring of strain in masonry panels tested under compression loads [38], (ii) the detection and localization of earthquake-induced damage to masonry buildings [39], and (iii) the strain field reconstruction and damage identification in masonry walls subjected to in-plane loading conditions [40]. An extensive investigation of the removal of the environmental effects from strain measured by smart bricks was also carried out [41].

This paper aims to investigate the strain-sensitivity of smart bricks when inserted in masonry walls subjected to in-plane shear loading through experimental tests and numerical simulations. The use of smart bricks for strain monitoring and crack detection in this typical structural setting represents a novelty. In addition, smart bricks are produced in standard dimensions, and were then adopted for the first time to construct and instrument real-scale masonry structural elements. The paper is organized as follows. Section 2 describes the smart bricks by briefly recalling their strain-sensing principle, preparation process, and the electromechanical model adopted to estimate strain measurements. Section 3 describes the tested masonry wall specimens, their instrumentation, and the test methodology. Section 4 outlines the approach that was considered to conduct the numerical simulations. Section 5 presents the obtained results, while Section 6 reports their discussion. Finally, Section 7 concludes the paper with comments and remarks.

## 2. Smart Bricks

This section recalls the meaningful concepts that form the basis of the smart brick technology that was used to monitor strain in real-scale masonry structural elements; considering this, the smart sensors were tailored, sized, and characterized for adoption as standard clay bricks during the construction of the tested masonry wall specimens.

### 2.1. Basic Sensing Principle

The strain-sensing capability of smart bricks derives from the contributions of the piezoresistivity of the doped clay matrices and the contact resistance developed at their electrodes [38]. However, the piezoresistive behavior mostly drives the sensing principle of these innovative sensors: this is enhanced by the tailored composition of the mix design of the smart bricks, which adds specific, electrically conductive fillers to the clay matrices. Leveraging the piezoresistive principle, smart bricks, when inserted in masonry structures, can be used to assess variations in strain by monitoring their electrical outputs, such as the electrical resistance. When deployed in coarse sensor networks, smart bricks can be used to monitor permanent changes in strain, e.g., changes due to earthquake-induced damage or differential settlements, by comparing their actual strain measurements with those referring to the undamaged structure [39]. Smart bricks’ strain outputs can be processed to reconstruct strain fields in masonry constructions/structural elements when the novel sensors are diffusely deployed within the load-bearing systems. In this circumstance, damage detection can be performed by seeking anomalies in the reconstructed strain field maps [37,40].

### 2.2. Materials and Preparation

**Figure 1:**
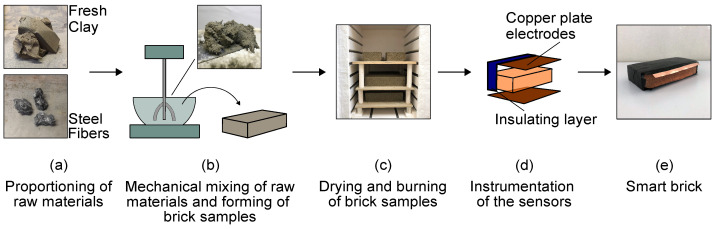
Production process of a sample of smart brick.

The geometrical dimensions of the smart bricks used in this experimentation were equal to those of the conventional clay bricks used to construct the masonry wall specimens described in Section 3.1, i.e., 250 × 55 × 120 mm${}^{3}$. The doped clay matrices that formed the smart bricks were obtained by adding stainless steel microfibers, the electrically conductive fillers, with fresh clay at a proportion of 0.25% by weight [38]. Specifically, the fibers were model R.STAT/S, with a diameter of 12 μm and length of 5 mm [42]. Figure 1 shows the principal phases of the preparation procedure of the smart bricks. First, clay and scattered steel microfibers were mechanically mixed to obtain a homogeneous composite with an optimal dispersion of the inclusions (Figure 1a). The resulting compound was then poured into wooden molds sprinkled with sand (Figure 1b), and fresh smart bricks were formed. The samples were dried at 90 °C for six hours, then burned at 900 °C for an additional six hours using a professional oven (Figure 1c). Once they had cooled down, burned bricks were first instrumented with two copper plates electrodes, applied at their upper and bottom sides, then covered with an insulating tape that protects the sensors from external electrical fields (Figure 1d). Finally, Figure 1e exemplifies a sample of smart brick.

### 2.3. Electromechanical Model of Smart Bricks

Smart bricks can exhibit changes in their electrical outputs while strained under compression. A specific electromechanical model, named the series resistors model, was developed by linearly combining the contributions to the strain-sensing capability due to the piezoresistive behavior and the contact resistance that develops at the electrodes of the sensors. The series resistor model, upon proper calibration, can be used to estimate strain measurements from the electrical outputs monitored from smart bricks as follows [38]:

(1) $$\frac{\Delta R}{R_{i,0}}=\frac{R-R_{i,0}}{R_{i,0}}≅a^{\prime }\epsilon^{-b}-\lambda \epsilon ,$$

where $a^{\prime }$ expresses the relative sensing at the interfaces between the surfaces of smart bricks and their electrodes, *b* is a positive constant referring to the contact resistance, $R_{i,0}$ represents the unstrained internal electrical resistance of the smart bricks, and λ is the gauge factor. This last parameter, in particular, characterizes the piezoresistive behavior of the smart bricks.

## 3. Experimental Application

This section describes the tested masonry wall specimens and their instrumentation. The experimental testing procedure is also outlined.

### 3.1. Masonry Wall Specimens

**Figure 2:**
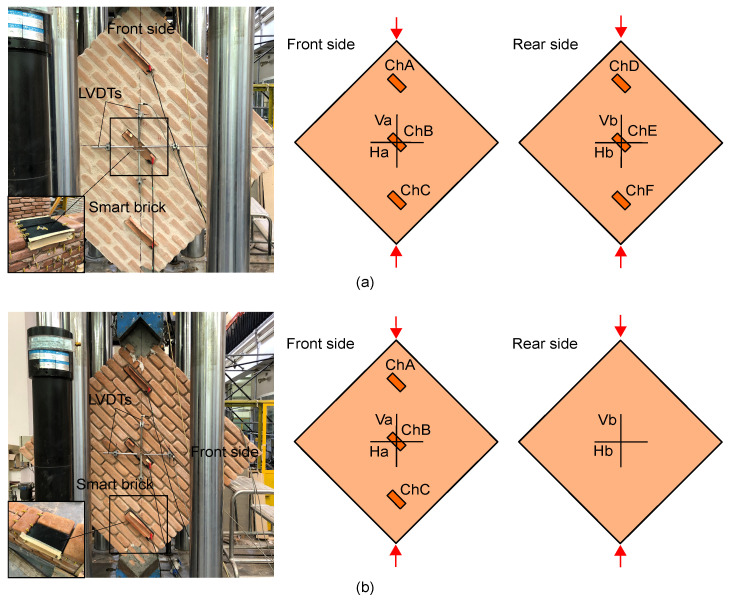
Pictures of the tested masonry wall specimens with the indication of the placement of smart bricks and LVDTs: (**a**) Specimen A with intact mortar layers; (**b**) Specimen B with degraded mortar layers.

Smart bricks’ effectiveness at measuring strain when inserted within masonry walls subjected to in-plane shear loading was investigated by testing two full-scale double-leaf masonry wall specimens, with different characteristics, under diagonal compression. Both the specimens had geometrical dimensions of 1290 × 250 × 1290 mm${}^{3}$, and were constructed by arranging conventional clay bricks with mortar layers of about 10 mm thickness. Specifically, specimen A had intact mortar layers and represented a masonry wall of recent construction. Specimen B was built with degraded mortar layers, with joints having 3/4 the planned thickness with respect to specimen A, to simulate the mechanical response of aged masonry walls (material degradation due to mortar aging). Smart bricks were embedded in both the masonry panels during the construction phase to monitor changes in strain. Figure 2 shows pictures of the wall specimens placed in the test configuration, together with the placement of the adopted sensing technologies, i.e., smart bricks and LVDTs. Smart bricks were inserted along the compressed diagonal of both the wall specimens, as they were conceived to monitor variations in compressive strain by instrumenting the front and rear sides of specimen A but only the front side of specimen B. The diversified use of smart bricks took into account the two possible application configurations of the novel sensors, i.e., on both sides of masonry walls in new constructions (represented by specimen A), or substituting a limited number of traditional bricks in existing structures (represented by specimen B). Hence, in most cases, masonry units in existing multi-leaf masonry walls can be replaced by smart bricks into a single masonry leaf to minimize the invasiveness of brick replacement operations. Both the masonry wall specimens were instrumented with LVDTs that were externally attached on both their front and rear sides to measure displacements that occurred parallel and orthogonal to the applied loads [43,44,45].

### 3.2. Diagonal Compression Tests

Diagonal compression tests were conducted under displacement control by applying displacements with increasing magnitude until failure of the wall specimens occurred. A multichannel approach [46], developed to simultaneously acquire strain measurements from an arbitrary number of independent smart sensors, was adopted to retrieve strain outputs from the smart bricks that were embedded in the masonry structural elements. Electrical measurements were conducted on smart bricks at increasing levels of the applied load. The novel sensors were supplied with a voltage square wave input of 20 V peak-to-peak, duty cycle of 50%, and frequency of 1 Hz; in the meantime, measurements of the voltage drop were carried out at the reference resistor, which was placed in series with each smart brick. This operation was conducted with a constant sampling rate of 10 Hz. The total electrical resistance of the *n*-th smart brick of the sensor network, $R_{n}$, was computed as follows:

(2) $$R_{n}=\frac{V-V_{\mathrm{drop},n}}{I_{n}},$$

where *V* is the positive value of the voltage input signal provided to each smart brick (+10 V), $V_{\mathrm{drop},n}$ is the voltage drop measured at 80% of the cyclic positive constant part of the voltage intensity output acquired at the reference resistor, and $I_{n}$ represents the current flowing within the *n*-th channel [47]. Strain measurements were retrieved from each smart brick through Equation (1), which was specialized using the terms collected in Table 1. These parameters were determined with a calibration procedure conducted in laboratory [38] that involved smart bricks before they were embedded in the wall specimens. For comparative purposes, strain measurements were also retrieved from the displacement outputs gathered from LVDTs through the following relations:

(3) $$\epsilon =\epsilon_{v}+\epsilon_{h},$$

(4) $$\epsilon_{v}=\frac{\Delta V_{a}+\Delta V_{b}}{2e},$$

(5) $$\epsilon_{h}=\frac{\Delta H_{a}+\Delta H_{b}}{2e},$$

where $\Delta V_{a}$ and $\Delta V_{b}$ represent the relative displacements measured in the front and rear sides of each wall specimen, respectively, parallel to the applied load; $\Delta H_{a}$ and $\Delta H_{b}$ denote the relative displacements measured at the front and rear sides of each wall specimen, respectively, along the direction orthogonal to the applied load; and *e* represents the baseline measurement of the LVDTs, which was equal to 400 mm.

## 4. Numerical Simulations

**Figure 3:**
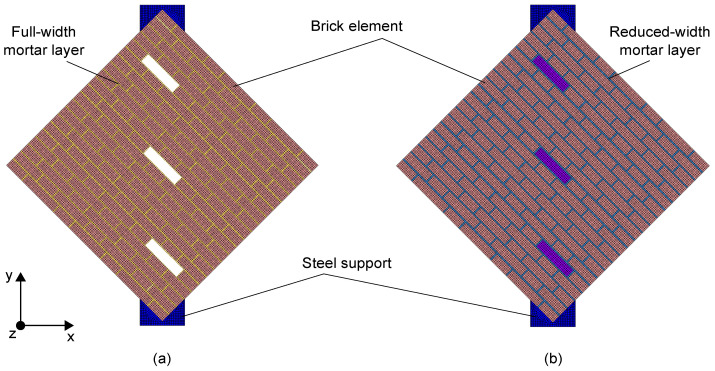
Numerical models: (**a**) Specimen A with intact mortar layers; (**b**) Specimen B with degraded mortar layers.

**Figure 4:**
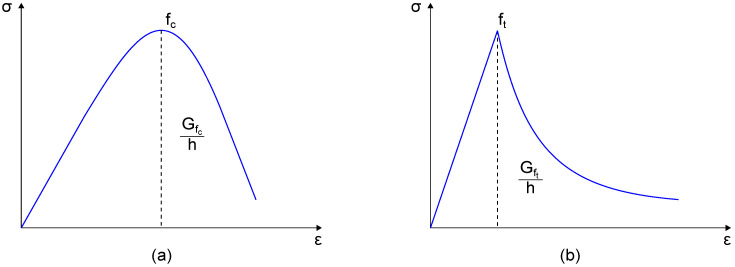
Mechanical behavior of brick elements and mortar layers: (**a**) stress–strain curve in compression; (**b**) stress–strain curve in tension.

The Finite Element Method (FEM) was used to simulate the mechanical response of the two masonry wall specimens tested under diagonal compression. Two-dimensional numerical models, shown in Figure 3, were built using the FEM software TNO DIANA [48] (version 10.4) under the assumption of plane-stress conditions. The 2D analysis represented a valid modeling strategy given the geometry of the wall specimens and the in-plane loading conditions. A simplified micro-modeling approach was used to separately model brick elements and mortar layers as continuous isotropic materials without contact interfaces. This modeling strategy can properly discretize the mechanical properties of both construction materials, as well as account for the presence of intact and degraded mortar layers between the two masonry wall specimens. This last feature was simulated by modeling full-width mortar layers in the numerical model of specimen A, while reduced-width joints (mortar joints with their width reduced by approximately 24%) were defined in the numerical model of specimen B. The modeling of smart bricks was neglected in both the numerical models to simulate the presence of mechanical weaknesses at the interface between the novel sensors and mortar layers. Although, during the mechanical tests, smart bricks demonstrated a resistance comparable to that of traditional clay bricks (no cracks occurred in smart bricks), some concerns arose regarding the mechanical interaction between the insulating tape covering smart bricks and mortar joints, as discussed in Section 6. Eight-node quadrilateral isoparametric curved shell elements (CQ40S) were used to discretize brick elements and mortar layers. The mesh size was equal to the thickness of the mortar layers and was selected after a mesh sensitivity analysis. Figure 4 reports the stress–strain response curves that were utilized to describe the mechanical behavior of both brick elements and mortar layers in the numerical simulations. The tensile behavior of the materials was assumed to be linear and elastic up to the peak strength; then, an exponential relationship was considered to model the post-elastic response [49,50]. A parabolic model [51] was used to describe the behavior of the materials under compression. The stress–strain response curves were specialized for each material considered in the simulations according to the mechanical properties collected in Table 2. Among these, Young’s modulus, *E*, compressive strength, $f_{c}$, tensile strength, $f_{t}$, and Poisson’s coefficient *v* were estimated from experimental tests, while the fracture energy in compression, $G_{f_{c}}$, and that in tension, $G_{f_{t}}$, was taken from the literature [52], and hence was manually tuned. Damage due to tensile cracking was modeled using a rotating smeared-crack model, in which the stress–strain relationship was evaluated in the principal directions of the strain vector, so the direction of the cracks changed according to the direction of the principal strain. Regarding boundary conditions, constraints were assigned at the base of both the numerical models to prevent horizontal and vertical translations. Simplified steel supports, with elastic mechanical properties, were also included in the numerical models to simulate the real load application areas. The simulation of the diagonal compression tests was performed through imposed numerical displacements, with increasing magnitude, in agreement with the experimental test procedure. The numerical solutions were carried out using the Newton–Raphson method (using the secant stiffness matrix), together with a line-search procedure, which was used to solve the corresponding nonlinear equations.

## 5. Results

This section outlines the experimental and numerical results obtained in the presented study.

### 5.1. Experimental Application

**Figure 5:**
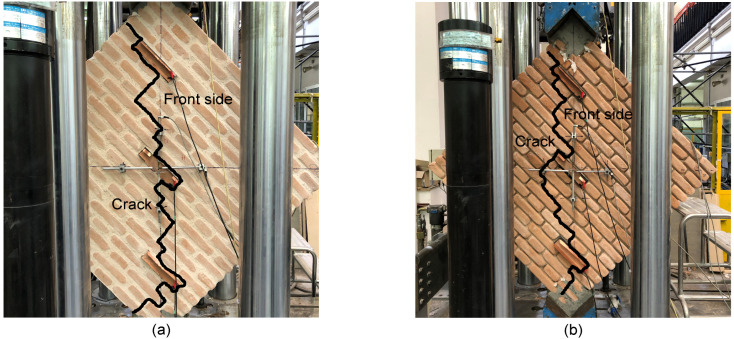
Cracking pattern detected on the tested wall specimens at the end of the tests: (**a**) Specimen A with intact mortar layers; (**b**) Specimen B with degraded mortar layers.

**Figure 6:**
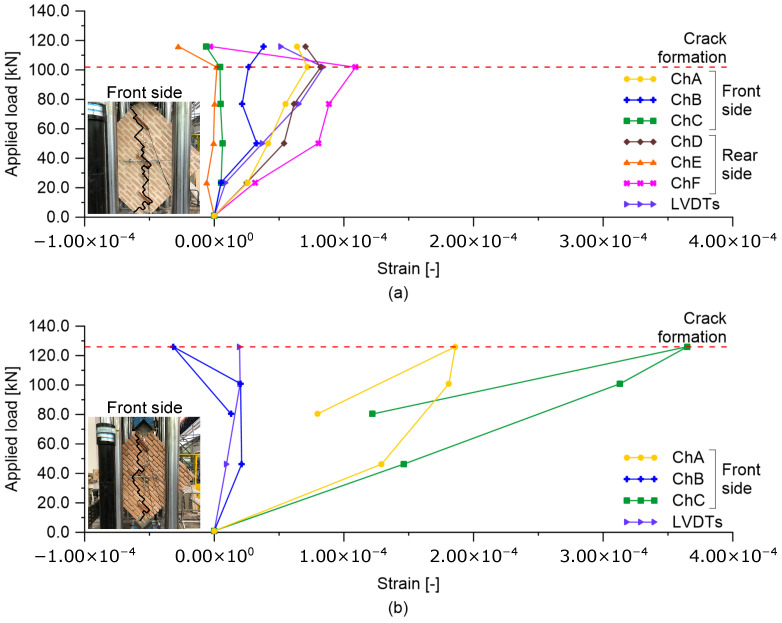
Trends of strain measured by smart bricks and LVDTs versus applied loads: (**a**) Specimen A with intact mortar layers; (**b**) Specimen B with degraded mortar layers.

Figure 5 shows the trends in the cracks developed on the masonry wall specimens tested under diagonal compression. The cracking patterns mainly formed in the compressed diagonal of the wall specimens, involving both the front and rear sides, with similar development paths. The thickness of the cracks was small at their formation to the extent that their initial identification was only possible through careful visual inspections. Specimen A cracked at a lower applied load value than specimen B, as shown in Figure 6, which also reports the strain measurements retrieved from smart bricks and LVDTs at different levels of the monitored load. Almost all the smart bricks within specimen A provided increasing compressive strains as the applied load increased (see Figure 6a). Then, at crack formation, around 100 kN, smart bricks revealed a marked decrease in their compressive strain states. The variation in the trends of the strain outputted by the novel sensors was, therefore, consistent with the cracking pattern developed on the tested specimen. It should be also noted that smart bricks in pairs, namely, sensors in channels A and D, B and E, and C and F, outputted comparable changes in strain when embedded in the same position on the wall specimen but at its front and rear side. In this regard, smart brick C may measure smaller strains than those of smart brick F due to its imperfect embedding within the double-leaf running-bond pattern, which led to a reduction in its strain-sensing ability. In addition, smart brick C may have been affected by a non-uniform redistribution of stresses within the wall specimen, which were perhaps caused by the uneven contact between the masonry and the lower steel support. Smart bricks were also able to detect the formation of a cracking pattern in specimen B at around 120 kN (see Figure 6b). In this circumstance, the novel sensors were affected by a quite sharp reduction in their compressive strain states when the cracks formed. In general, smart bricks positioned at the edges of the compressed diagonal of both specimens were more stressed in compression than the smart sensors placed in the middle areas of the structural elements. Furthermore, smart bricks embedded in specimen B were subjected to higher compressive strain states than smart bricks instrumenting specimen A. This is because, in the latter case, with intact mortar layers, compressive stresses were more evenly distributed in the structural components. Strain measurements retrieved from LVDTs were comparable in terms of trend and magnitude with the outputs from the smart bricks positioned in the middle of the compressed diagonal of both the tested specimens. Note that, in Figure 6b, no data are available for LVDTs after the formation of the cracking pattern. In fact, LVDTs were removed from specimen B to prevent them from being damaged by a potential brittle failure of the structural element due to the presence of degraded mortar layers.

### 5.2. Numerical Simulations

**Figure 7:**
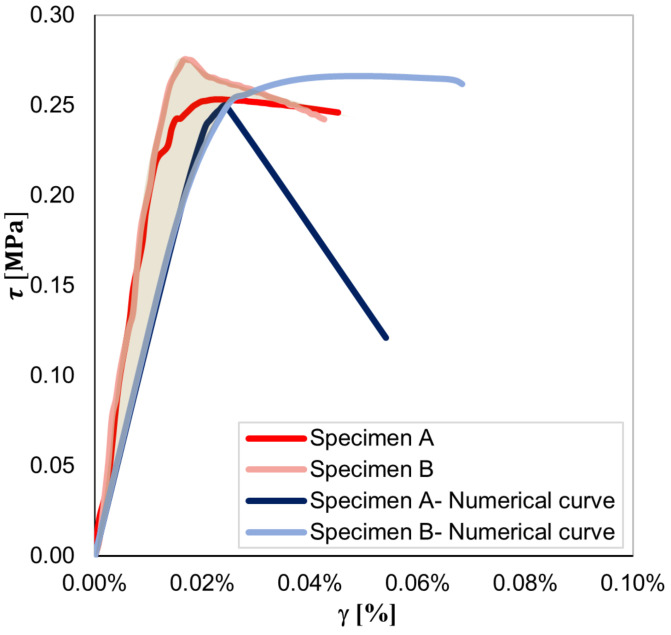
Numerical versus experimental shear stress–strain curves obtained for the tested masonry wall specimens.

**Figure 8:**
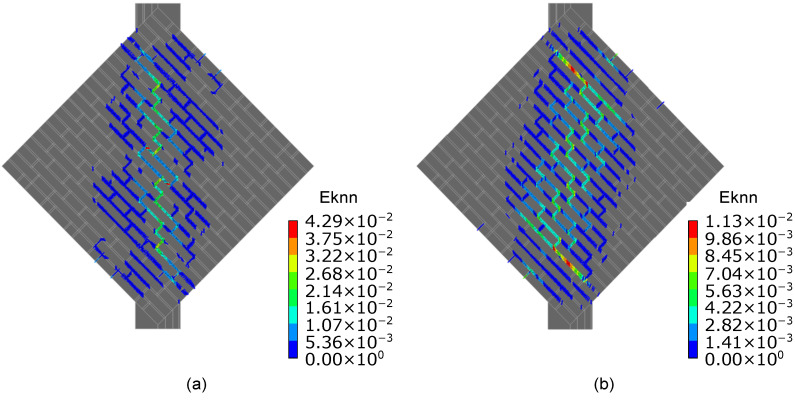
Cracking pattern obtained from the numerical simulations (Eknn denotes the normal crack strain): (**a**) Specimen A with intact mortar layers; (**b**) Specimen B with degraded mortar layers.

**Figure 9:**
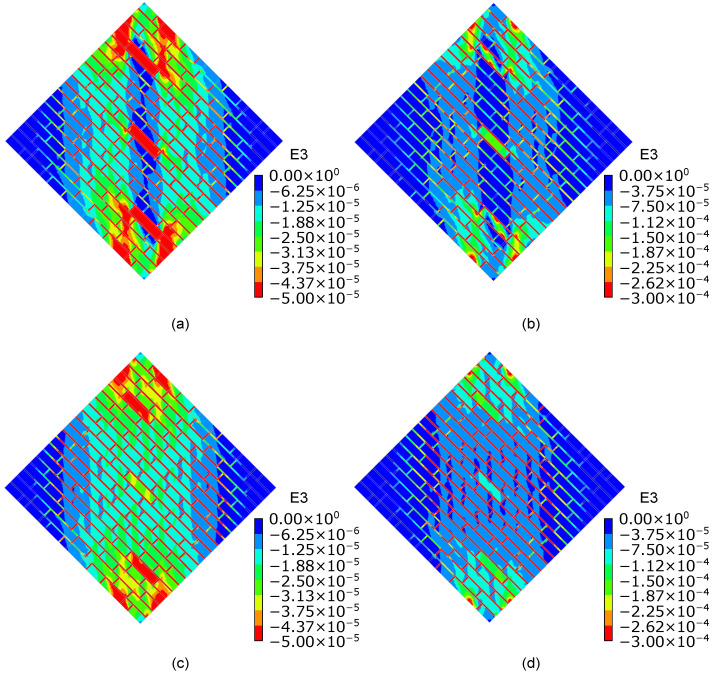
Compressive strain fields obtained from the numerical simulations (E3 denotes the compressive principal strain): (**a**) Strain state of specimen A at the one-third of peak shear stress; (**b**) strain state of specimen A at the peak shear stress; (**c**) strain state of specimen B at the one-third of peak shear stress; (**d**) strain state of specimen B at the peak shear stress.

Figure 7 shows a satisfactory agreement between the numerical and experimental shear stress–strain curves until peak conditions are reached. Experimentally, a peak shear stress equal to 0.25 and 0.27 MPa was determined for specimens A and B, respectively. Consistently, the numerical model simulating the mechanical response of specimen A outputted a lower peak shear stress than that of specimen B. The post-peak response of both numerical models differed from that observed experimentally. In the case of specimen A, the sharp drop visible in the softening branch of the numerical shear stress–strain curve can be attributed to the decision to neglect smart bricks in the numerical simulations and the assumption of a reduced cross-section of the numerical model in areas in which novel sensors were embedded. Figure 8 details the cracking patterns that were numerically found for specimens A and B. The obtained results are quite consistent with the cracking patterns illustrated in Figure 5. Both numerical and experimental cracks were concentrated on the compressed diagonal of the wall specimens, even if a more evident diffuse cracking pattern resulted from the numerical simulations carried out for specimen B. Figure 9 illustrates the compressive strain fields at one-third of peak shear stress (before crack formation) and at the peak shear stress (at crack formation) obtained for both the specimens from the numerical simulations. Prior to the formation of the cracking patterns, strains were evenly distributed in the compressed diagonal of the numerical models, affecting both brick elements and mortar layers. However, the structural components at the edges of the compressed diagonals were the most strained. Then, at crack formation, the higher strain values were mainly located at the edges of the compressed diagonals and at the mortar layers, while strains of reduced entities affected the brick elements comprising the central regions of both the numerical models. The numerical simulations, therefore, pointed out that compressive strains migrated from the central regions of the numerical models toward the load application areas at crack formation.

## 6. Discussion

The results obtained by testing the masonry wall specimens under diagonal compression loads demonstrated the effectiveness of smart bricks at measuring strain when inserted within masonry walls subjected to in-plane shear loading. In addition, the experiments also showed that smart bricks could detect the formation of shear-induced damage at a very early stage of development, by demonstrating marked changes in their strain outputs corresponding to crack initiation. Despite the differences between the sensing principle of smart bricks and LVDTs, the comparison of these two sensing technologies denoted good consistency between their strain outputs. In fact, strain measurements from the smart bricks that were centrally positioned in the wall specimens and corresponding LVDTs were comparable both in terms of trend and magnitude. A satisfactory agreement was also found between the experimental results and those obtained from numerical simulations until peak conditions were reached. Experimentally, the presence of smart bricks at both the front and rear sides of specimen A may have influenced the shear response of the wall specimen. Indeed, specimen A, with intact mortar layers, cracked at a lower shear stress than specimen B, which simulated an aged masonry panel with degraded mortar joints. This unexpected mechanical behavior may be caused by the mechanical weaknesses that developed at the contact interfaces between mortar layers and the insulating tape covering the smart bricks. As the novel sensors were embedded in pairs within the thickness of specimen A, this masonry panel may have been more affected by the supposed weak contact between smart bricks and mortar layers compared to specimen B. Nevertheless, given the limited number of tested wall specimens, it is not impossible that the obtained experimental findings may be due to the statistical variability of the results. Considering this, further investigations would be necessary to characterize the interface between smart bricks and mortar layers under shear stress conditions. In a first approximation, the FE models of the wall specimens were constructed assuming the presence of mechanical weaknesses at the interface between smart bricks and mortar layers. The numerical models were able to simulate the mechanical response of the wall specimens until the achievement of peak conditions, as demonstrated by comparing experimental and numerical shear stress–strain curves, but misinterpreted their post-peak behavior. This was particularly evident in the numerical simulations that were carried out for specimen A. However, numerical models are reliable when interpreting the strain measurements outputted by smart bricks up to peak conditions. In confirmation of this, it is worth emphasizing that the numerical analyses showed higher strain concentrations at the edges of the compressed diagonals of the tested wall specimens with respect to their central areas. Similar results were experimentally determined through the smart bricks’ outputs. For instance, the smart bricks positioned at the edges of the compressed diagonal of specimen B were more strained in compression than the smart bricks placed in the middle area of the structural element. In addition, at peak condition/crack formation, numerical models provided strain values in the proximity of the smart bricks’ positions that were of a comparable magnitude to those outputted experimentally by the novel sensors, i.e., approximately equal to $10^{-4}$. Overall, the outcomes of the numerical simulations help in the interpretation of both the trend and extent of the strain measurements provided by smart bricks up to peak conditions.

## 7. Conclusions

The paper proposed an experimental investigation of the effectiveness of smart bricks of standard dimensions in strain monitoring and crack detection when inserted within masonry walls subjected to in-plane shear loading. Numerical simulations were also carried out to interpret the experimental outcomes.

The main concepts behind the smart brick technology were recalled by presenting the strain-sensing principle and production process of the novel clay-based sensors. Then, the paper presented an experimental program that was carried out on two masonry wall specimens instrumented with full-scale embedded smart bricks and LVDTs. The methodology that was adopted to test the structural elements under diagonal compression was described, together with the approaches adopted to retrieve strain measurements from smart bricks and LVDTs. Details related to the construction of the two-dimensional FE models used to numerically simulate the performed experiments were also provided.

Almost all the smart bricks outputted an increasing compressive strain as the load applied on the tested wall specimens increased. Furthermore, the novel sensors provided variations in the trend of their strain outputs that were clearly attributable to crack initiation in wall specimens. A good agreement in terms of strain magnitude and trend was found by comparing smart brick and LVDT outputs. Limited discrepancies were observed, which can reasonably be attributed to the different nature of these sensing technologies. Numerical models were able to interpret the mechanical response of the wall specimens up to the formation of cracking patterns in the structural elements. This was demonstrated by comparing the experimental and numerical shear stress–strain curves. Numerical simulations were used to interpret the trend and extent of the strain measurements provided by smart bricks up to crack formation. A more than acceptable agreement was found, even under this circumstance, between numerical and experimental results.

Overall, the experimental and numerical outcomes obtained in this research work demonstrate that smart bricks of standard dimensions can be used for strain monitoring and early crack detection in masonry structural elements subjected to in-plane shear loading. These monitoring capabilities make the smart brick technology an attractive approach for the SHM of masonry constructions. This is furthered by the well-known susceptibility of masonry structures, especially existing ones, to shear-induced damage. However, as anticipated in Section 6, further investigations are needed to mechanically characterize the interface between smart bricks and mortar layers under shear stress conditions. In addition, the embedding of smart bricks in existing masonry structural elements should be experimentally investigated, as, in this work, the use of novel sensors was limited to a structural setting simulating the mechanical response of aged masonry walls. To conclude, considering the state-of-the-art and the evidence from the proposed research work, the smart brick technology appears mature enough to be employed in exploratory SHM applications involving full-scale masonry buildings subjected to realistic loading and environmental conditions.

## Figures and Tables

**Table 1:** Parameters used to specialize Equation (1) to retrieve strain measurements from the smart bricks embedded within the tested masonry wall specimens: $R_{i,0}$ represents the unstrained internal electrical resistance of the smart bricks, $a^{\prime }$ is the relative sensing at the contact interfaces between the surfaces of the sensors and their electrodes, *b* represents a positive constant referring to the contact resistance, and λ is the gauge factor.

	$R_{i,0}$ [MΩ]	$a^{\prime }$	*b*	λ
Smart bricks	19.6	$4.70\times 10^{-8}$	2.16	1603

**Table 2:** Mechanical properties considered in the numerical simulations. For every material, *E* is the elastic modulus, $f_{c}$ represents the compression strength, $f_{t}$ is the tensile strength, $G_{f_{c}}$ expresses the fracture energy in compression, $G_{f_{t}}$ is the fracture energy in tension, and *v* is the Poisson’s coefficient.

Material	*E* [MPa]	$f_{c}$ [MPa]	$f_{t}$ [MPa]	$G_{f_{c}}$ [Nmm]	$G_{f_{t}}$ [Nmm]	*v* [-]
Brick	13000	20.79	5.90	$6.60\times 10^{-1}$	$8.50\times 10^{-2}$	0.2
Mortar	1500	2.52	0.77	$5.60\times 10^{-2}$	$3.00\times 10^{-3}$	0.2

## Data Availability

The data that support the findings of this study are available from the authors upon reasonable request.

## References

[1] Glisic B., Hubbell D.L., Sigurdardottir D.H., Yao Y. (2013). Damage detection and characterization using long-gauge and distributed fiber optic sensors. Opt. Eng..

[2] Han B., Ding S., Yu X. (2015). Intrinsic self-sensing concrete and structures: A review. Measurement.

[3] Ascione F., Ceroni F., De Masi R.F., de’Rossi F., Pecce M.R. (2017). Historical buildings: Multidisciplinary approach to structural/energy diagnosis and performance assessment. Appl. Energy.

[4] Masciotta M.G., Pellegrini D., Brigante D., Barontini A., Lourenço P.B., Girardi M., Padovani C., Fabbrocino G. (2020). Dynamic characterization of progressively damaged segmental masonry arches with one settled support: Experimental and numerical analyses. Frat. Integrita Strutt..

[5] Augenti N., Parisi F. (2010). Learning from construction failures due to the 2009 L’Aquila, Italy, earthquake. J. Perform. Constr. Facil..

[6] Parisi F., Lignola G.P., Augenti N., Prota A., Manfredi G. (2013). Rocking response assessment of in-plane laterally-loaded masonry walls with openings. Eng. Struct..

[7] Saisi A., Gentile C., Ruccolo A. (2018). Continuous monitoring of a challenging heritage tower in Monza, Italy. J. Civ. Struct. Health Monit..

[8] Parisi F., Augenti N. (2013). Earthquake damages to cultural heritage constructions and simplified assessment of artworks. Eng. Fail. Anal..

[9] Fiorentino G., Forte A., Pagano E., Sabetta F., Baggio C., Lavorato D., Nuti C., Santini S. (2018). Damage patterns in the town of Amatrice after August 24th 2016 Central Italy earthquakes. Bull. Earthq. Eng..

[10] Dolce M., Prota A., Borzi B., da Porto F., Lagomarsino S., Magenes G., Moroni C., Penna A., Polese M., Speranza E. (2021). Seismic risk assessment of residential buildings in Italy. Bull. Earthq. Eng..

[11] Ludeno G., Cavalagli N., Ubertini F., Soldovieri F., Catapano I. (2020). On the combined use of ground penetrating radar and crack meter sensors for structural monitoring: Application to the historical Consoli Palace in Gubbio, Italy. Surv. Geophys..

[12] García-Macías E., Ubertini F. (2022). Least Angle Regression for early-stage identification of earthquake-induced damage in a monumental masonry palace: Palazzo dei Consoli. Eng. Struct..

[13] Ivorra S., Pallarés F.J. (2006). Dynamic investigations on a masonry bell tower. Eng. Struct..

[14] Gentile C., Saisi A. (2007). Ambient vibration testing of historic masonry towers for structural identification and damage assessment. Constr. Build. Mater..

[15] Ramos L.F., Masciotta M., Lourenço P.B., Vasta M. SHM of a masonry chimney after a lightning accident. Proceedings of the 9th International Workshop on Structural Health Monitoring.

[16] Formisano A., Di Lorenzo G., Krstevska L., Landolfo R. (2021). Fem model calibration of experimental environmental vibration tests on two churches hit by L’Aquila earthquake. Int. J. Archit. Herit..

[17] Ashayeri I., Biglari M., Formisano A., D’Amato M. (2021). Ambient vibration testing and empirical relation for natural period of historical mosques. Case study of eight mosques in Kermanshah, Iran. Constr. Build. Mater..

[18] Ahmadi S.S., Karanikoloudis G., Mendes N., Illambas R., Lourenço P.B. (2022). Appraising the Seismic Response of a Retrofitted Adobe Historic Structure, the Role of Modal Updating and Advanced Computations. Buildings.

[19] Civera M., Mugnaini V., Zanotti Fragonara L. (2022). Machine learning-based automatic operational modal analysis: A structural health monitoring application to masonry arch bridges. Struct. Control. Health Monit..

[20] Shimpi V., Sivasubramanian M.V., Singh S. (2022). Present day status and numerical modelling of heritage masonry bridges of Kalka-Shimla Mountain Railways. Int. J. Mason. Res. Innov..

[21] Borlenghi P., Saisi A., Gentile C. (2023). ND testing and establishing models of a multi-span masonry arch bridge. J. Civ. Struct. Health Monit..

[22] Derakhshan H., Visintin P., Griffith M.C. (2017). Case studies of material properties of late nineteenth-century unreinforced masonry buildings in Adelaide. Aust. J. Civ. Eng..

[23] Acikgoz S., Luciano A., Dewhirst M., Dejong M.J., Mair R. (2022). Innovative monitoring of the response of a heritage masonry building to nearby tunnelling in London Clay. Géotechnique.

[24] Nalon G.H., Ribeiro J.C.L., Pedroti L.G., da Silva R.M., de Araújo E.N.D., Santos R.F., de Lima G.E.S. (2022). Review of recent progress on the compressive behavior of masonry prisms. Constr. Build. Mater..

[25] Dalgic K.D., Gulen B., Liu Y., Acikgoz S., Burd H., Marasli M., Ilki A. (2023). Masonry buildings subjected to settlements: Half-scale testing, detailed measurements, and insights into behaviour. Eng. Struct..

[26] Pascariello M.N., Luciano A., Bilotta E., Acikgoz S., Mair R. (2023). Numerical modelling of the response of two heritage masonry buildings to nearby tunnelling. Tunn. Undergr. Space Technol..

[27] Tang Y., Chen M., Lin Y., Huang X., Huang K., He Y., Li L. (2020). Vision-based three-dimensional reconstruction and monitoring of large-scale steel tubular structures. Adv. Civ. Eng..

[28] Shamsabadi E.A., Xu C., Dias-da Costa D. (2022). Robust crack detection in masonry structures with Transformers. Measurement.

[29] Mousavi M., Bakhshi A. (2022). Crack detection in masonry structures using computer vision based on deep learning. Sharif J. Civ. Eng..

[30] Sangirardi M., Altomare V., De Santis S., de Felice G. (2022). Detecting damage evolution of masonry structures through computer-vision-based monitoring methods. Buildings.

[31] Tang Y., Zhu M., Chen Z., Wu C., Chen B., Li C., Li L. (2022). Seismic performance evaluation of recycled aggregate concrete-filled steel tubular columns with field strain detected via a novel mark-free vision method. Structures.

[32] Que Y., Dai Y., Ji X., Leung A.K., Chen Z., Tang Y., Jiang Z. (2023). Automatic classification of asphalt pavement cracks using a novel integrated generative adversarial networks and improved VGG model. Eng. Struct..

[33] Valvona F., Toti J., Gattulli V., Potenza F. (2017). Effective seismic strengthening and monitoring of a masonry vault by using Glass Fiber Reinforced Cementitious Matrix with embedded Fiber Bragg Grating sensors. Compos. Part B Eng..

[34] Verstrynge E., De Wilder K., Drougkas A., Voet E., Van Balen K., Wevers M. (2018). Crack monitoring in historical masonry with distributed strain and acoustic emission sensing techniques. Constr. Build. Mater..

[35] Barsocchi P., Bartoli G., Betti M., Girardi M., Mammolito S., Pellegrini D., Zini G. (2021). Wireless sensor networks for continuous structural health monitoring of historic masonry towers. Int. J. Archit. Herit..

[36] Downey A., D’Alessandro A., Laflamme S., Ubertini F. (2017). Smart bricks for strain sensing and crack detection in masonry structures. Smart Mater. Struct..

[37] García-Macías E., Ubertini F. (2019). Earthquake-induced damage detection and localization in masonry structures using smart bricks and Kriging strain reconstruction: A numerical study. Earthq. Eng. Struct. Dyn..

[38] Meoni A., D’Alessandro A., Ubertini F. (2020). Characterization of the strain-sensing behavior of smart bricks: A new theoretical model and its application for monitoring of masonry structural elements. Constr. Build. Mater..

[39] Meoni A., D’Alessandro A., Cavalagli N., Gioffré M., Ubertini F. (2019). Shaking table tests on a masonry building monitored using smart bricks: Damage detection and localization. Earthq. Eng. Struct. Dyn..

[40] Meoni A., D’Alessandro A., Kruse R., De Lorenzis L., Ubertini F. (2021). Strain field reconstruction and damage identification in masonry walls under in-plane loading using dense sensor networks of smart bricks: Experiments and simulations. Eng. Struct..

[41] Meoni A., Fabiani C., D’Alessandro A., Pisello A., Ubertini F. (2022). Strain-sensing smart bricks under dynamic environmental conditions: Experimental investigation and new modeling. Constr. Build. Mater..

[42] D’Alessandro A., Meoni A., Ubertini F. (2018). Stainless Steel Microfibers for Strain-Sensing Smart Clay Bricks. J. Sens..

[43] Corradi M., Borri A., Vignoli A. (2003). Experimental study on the determination of strength of masonry walls. Constr. Build. Mater..

[44] Borri A., Castori G., Corradi M. (2015). Determination of shear strength of masonry panels through different tests. Int. J. Archit. Herit..

[45] Celano T., Ceroni F., Lignola G.P. (2021). Behaviour of masonry walls strengthened with fibre-reinforced cementitious materials. Eng. Comput. Mech..

[46] Meoni A., D’Alessandro A., Mancinelli M., Ubertini F. (2021). A Multichannel Strain Measurement Technique for Nanomodified Smart Cement-Based Sensors in Reinforced Concrete Structures. Sensors.

[47] Downey A., D’Alessandro A., Ubertini F., Laflamme S., Geiger R. (2017). Biphasic DC measurement approach for enhanced measurement stability and multi-channel sampling of self-sensing multi-functional structural materials doped with carbon-based additives. Smart Mater. Struct..

[48] Chai S. (2020). Finite Element Analysis for Civil Engineering with DIANA Software.

[49] Hordijk D.A. (1991). Local Approach to Fatigue of Concrete. Ph.D. Thesis.

[50] Lignola G.P., Prota A., Manfredi G. (2009). Nonlinear analyses of tuff masonry walls strengthened with cementitious matrix-grid composites. J. Compos. Constr..

[51] Mininno G., Ghiassi B., Oliveira D.V. (2017). Modelling of the in-plane and out-of-plane performance of TRM-strengthened masonry walls. Key Eng. Mater..

[52] Lourenço P.B. (2010). Recent advances in masonry modelling: Micromodelling and homogenisation. Multiscale Model. Solid Mech. Comput. Approaches.

